# Supplementary material for: Air pollution control strategies directly limiting national health damages in the US
Source: Nat Commun. 2020 Feb 19;11:957. doi: 10.1038/s41467-020-14783-2 (PMC7031358; doi:10.1038/s41467-020-14783-2)
Supplement: Supplementary file 1 — Supplementary Information [file 41467_2020_14783_MOESM1_ESM.pdf]

# Air pollution control strategies directly limiting national health damages in the US

Ou et al.

**Supplementary Information**

**Supplementary Figures**

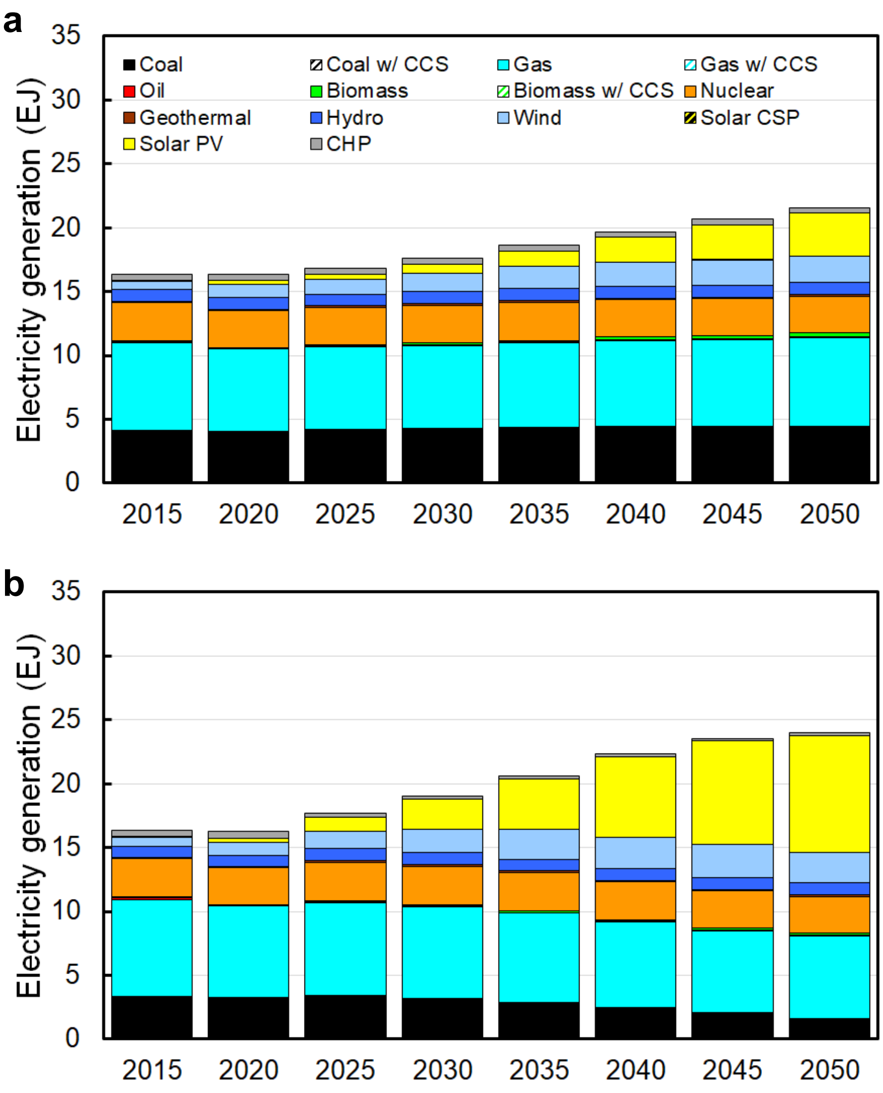


Supplementary Figure 1 Electricity generation by technology (EJ) in BASE REF (a) and HR REF (b).

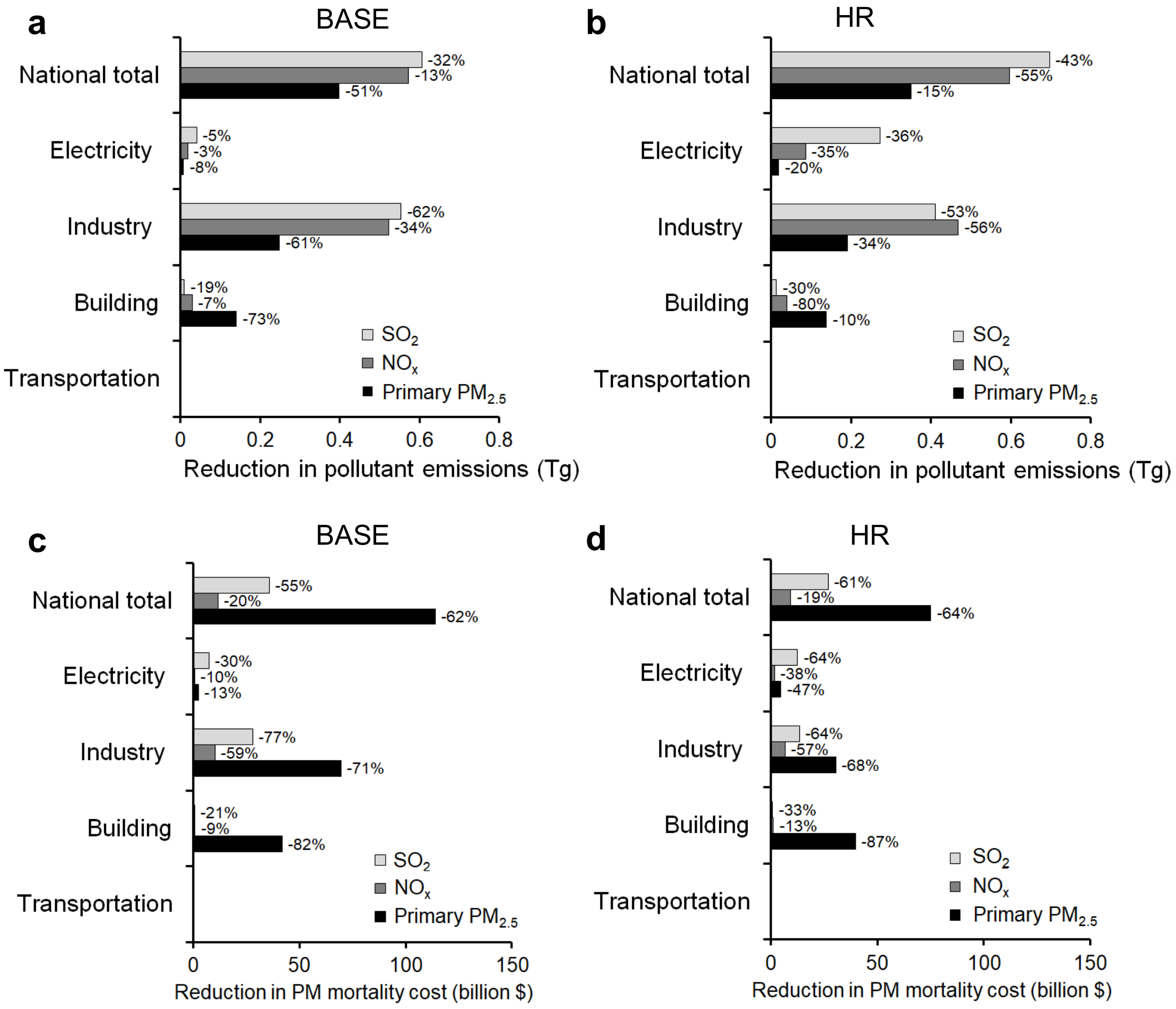


Supplementary Figure 2 **National air pollutant emissions and $PM_{2.5}$ mortality cost responses to the 50% $PM_{2.5}$ mortality cost reduction (US50) under two future energy trajectories**. Sectoral changes in emissions (Tg) by pollutant under BASE trajectory (a) and HR trajectory (b); sectoral changes in $PM_{2.5}$ mortality costs (billion $) by pollutant under BASE trajectory (c) and HR trajectory (d). Reductions are relative to the REF of the corresponding energy trajectory in 2050. Changes in the transportation sector are negligible.

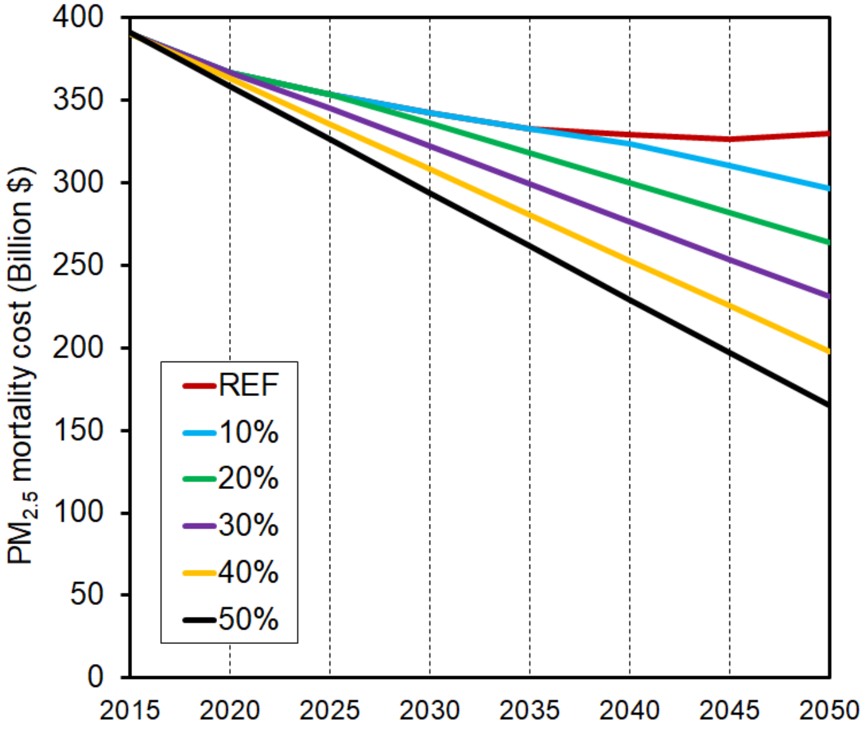


Supplementary Figure 3 Projected $PM_{2.5}$ mortality cost (PMMC) for REF and US10-US50 from 2015 to 2050.

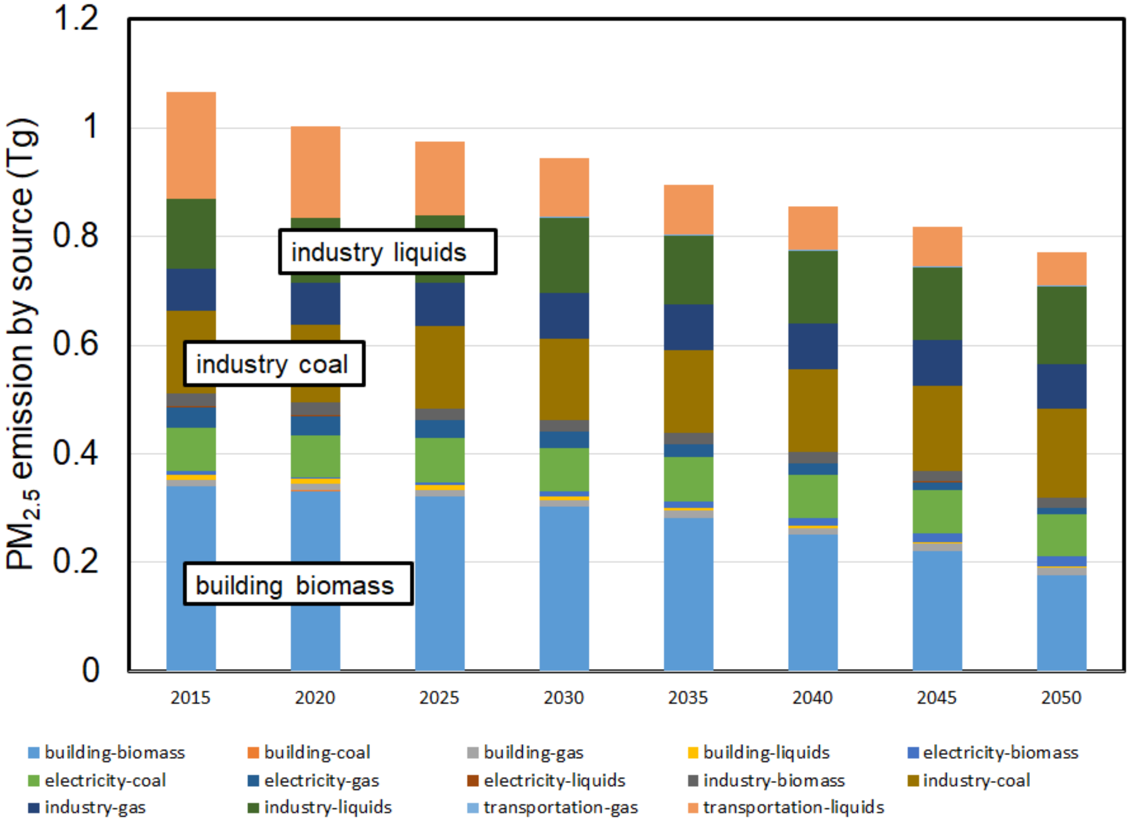


Supplementary Figure 4 Primary $PM_{2.5}$ emission by source sectors in REF (Tg).

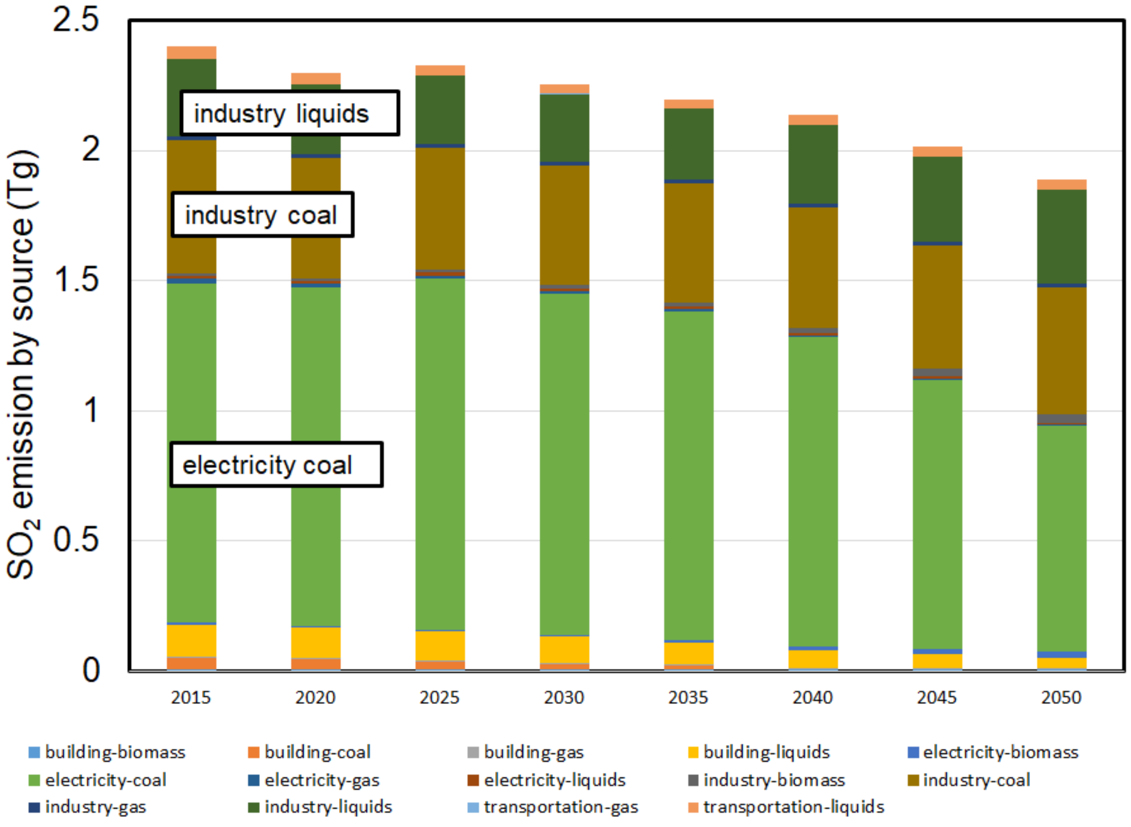


Supplementary Figure 5 $SO_2$ emission by source sectors in REF (Tg).

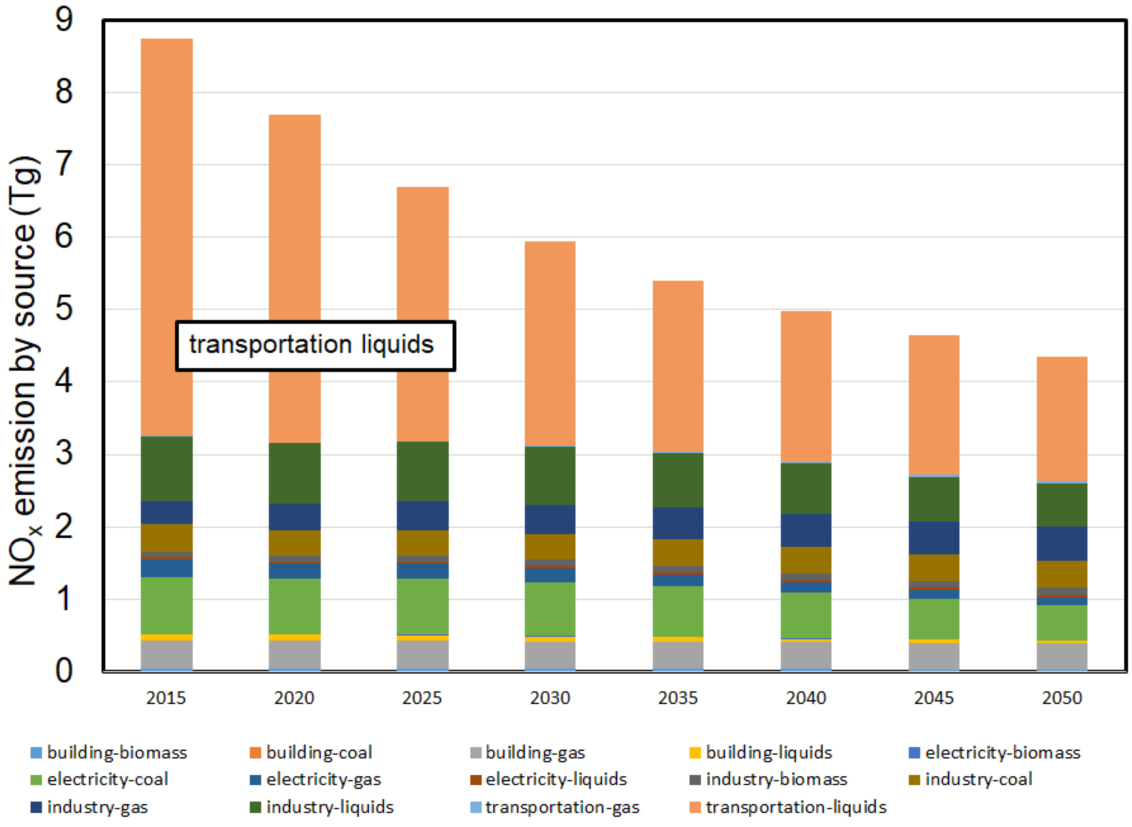


Supplementary Figure 6 $NO_x$ emission by source sectors in REF (Tg).

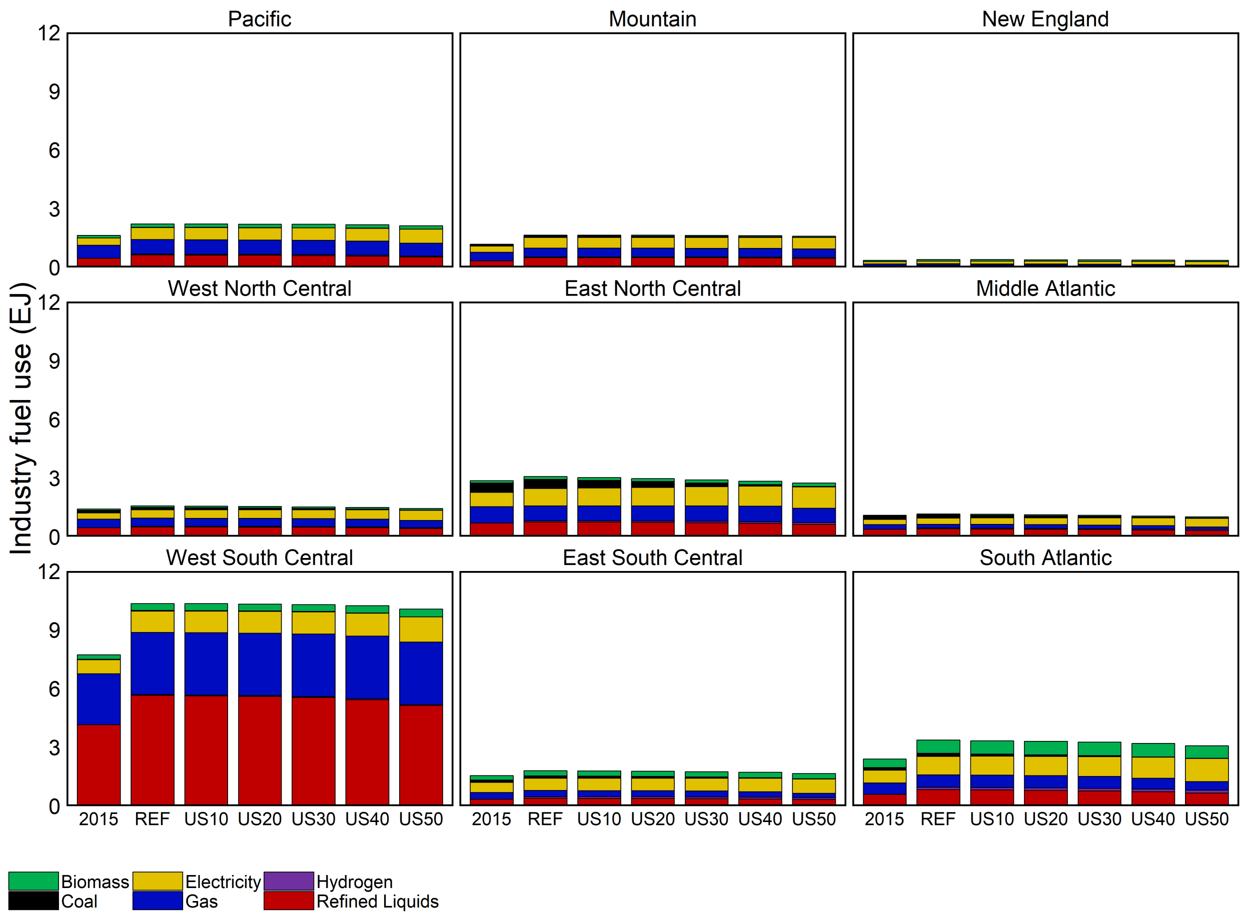


Supplementary Figure 7 Industrial fuel use for nine census regions in 2050 (except for the leftmost bar in each panel).

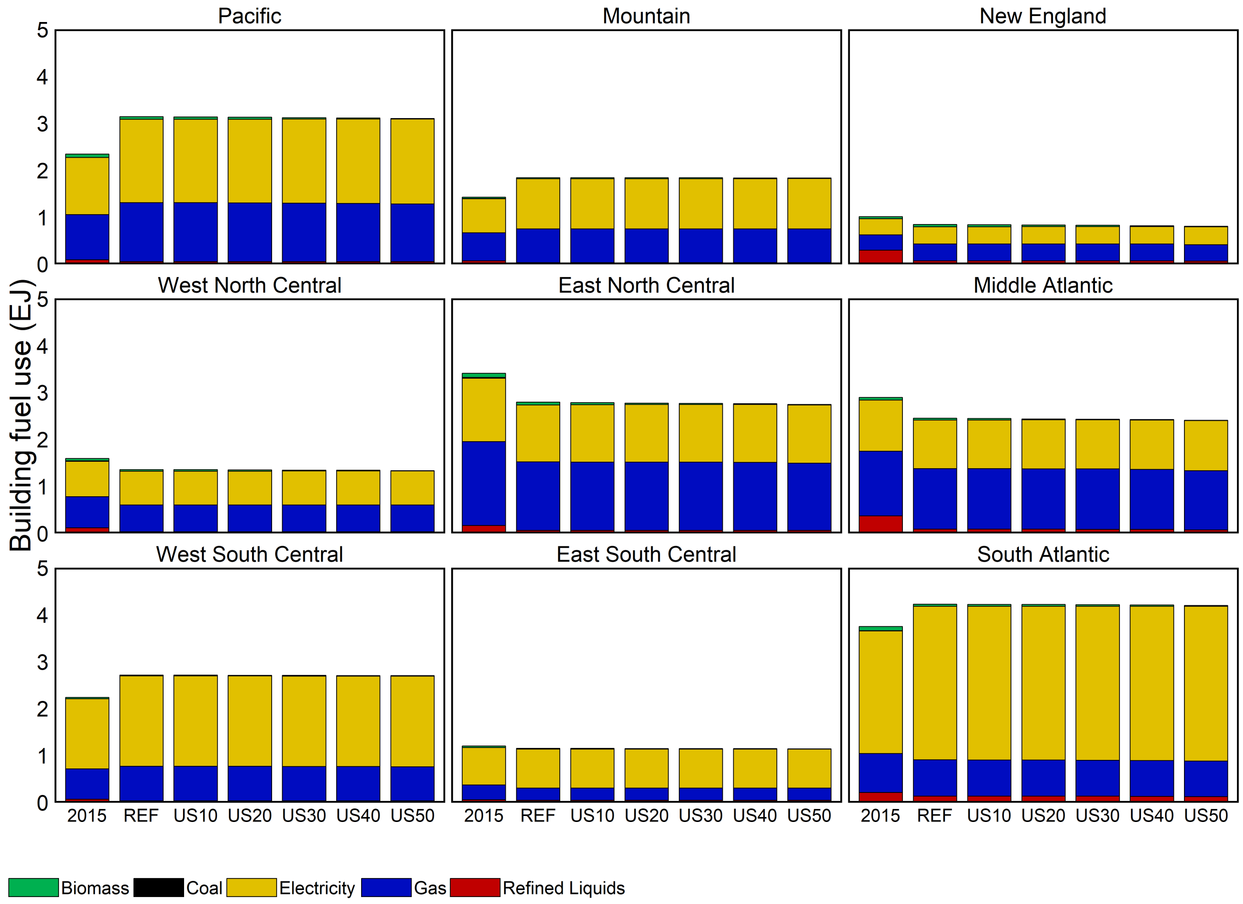


Supplementary Figure 8 Building fuel use (EJ) for nine census regions in 2050 (except for the leftmost bar in each panel).

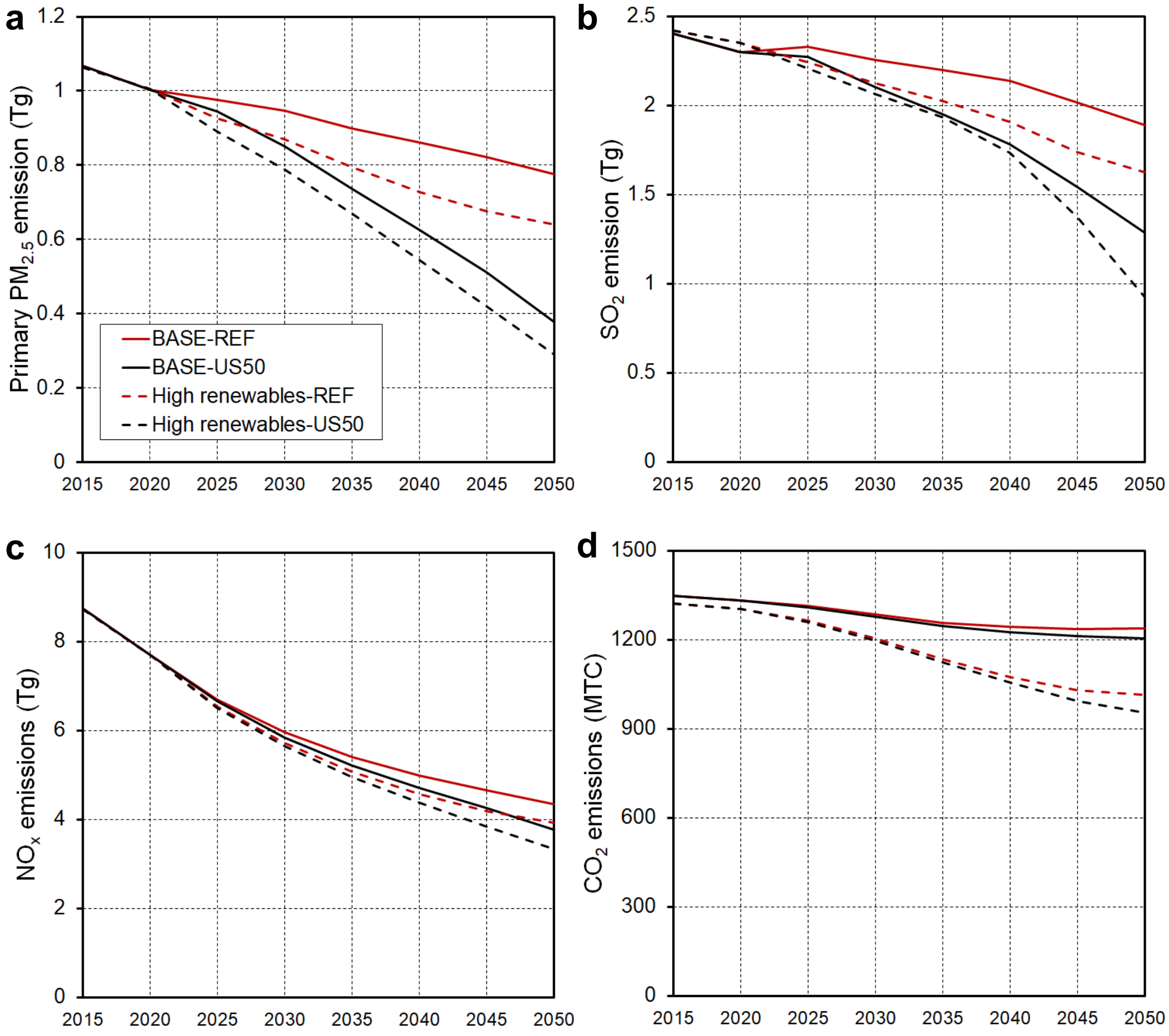


Supplementary Figure 9 Effects of $PM_{2.5}$ reduction stringency on national emissions of primary $PM_{2.5}$ (a), $SO_2$ (b), $NO_x$ (c), and $CO_2$ (d) in BASE and HR scenarios.

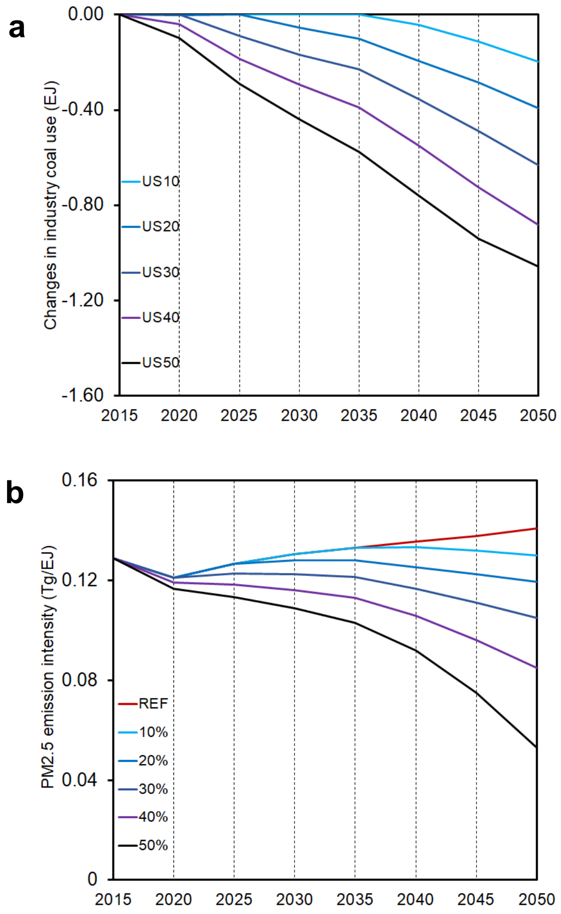


Supplementary Figure 10 Effects of PMMC constraint stringency on technology replacement. Changes in industrial coal use (EJ) (a), and average $PM_{2.5}$ emission intensity (Tg $EJ^{-1}$) of industrial coal (b), relative to REF2050 for each modeling year.

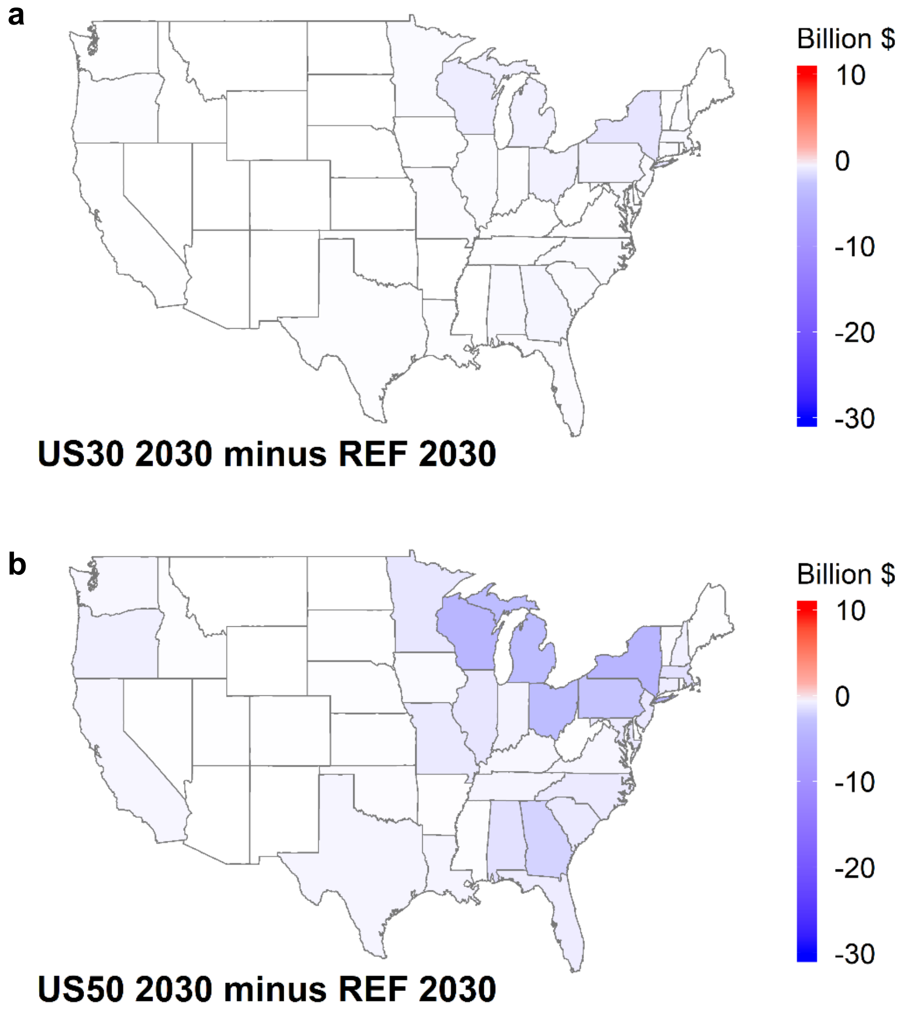


Supplementary Figure 11 Changes of state-level $PM_{2.5}$ mortality costs (2018$ billion) relative to REF for US30 in 2030 (a) and US50 in 2050 (b).

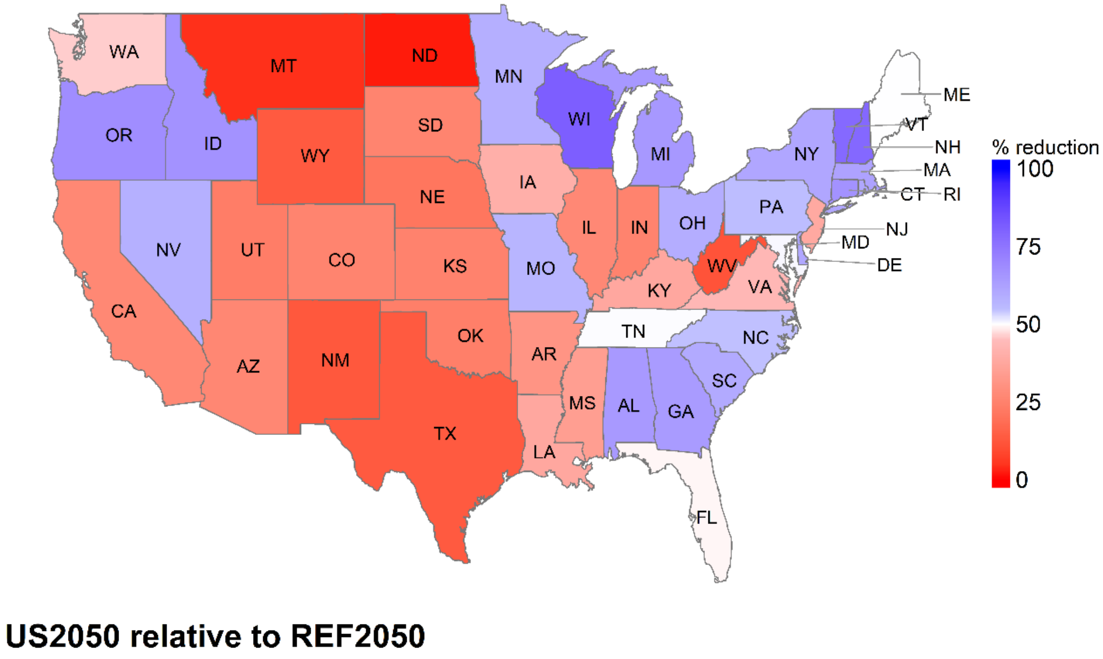


Supplementary Figure 12 Percentage reduction of PMMC in US50 relative to the REF in 2050 for each state. The color bar chooses 50% (the national target) as the benchmark: states in blue indicate greater percentage reductions for these states than the national percentage reduction target, and states in red indicate less percentage reductions then the national target.

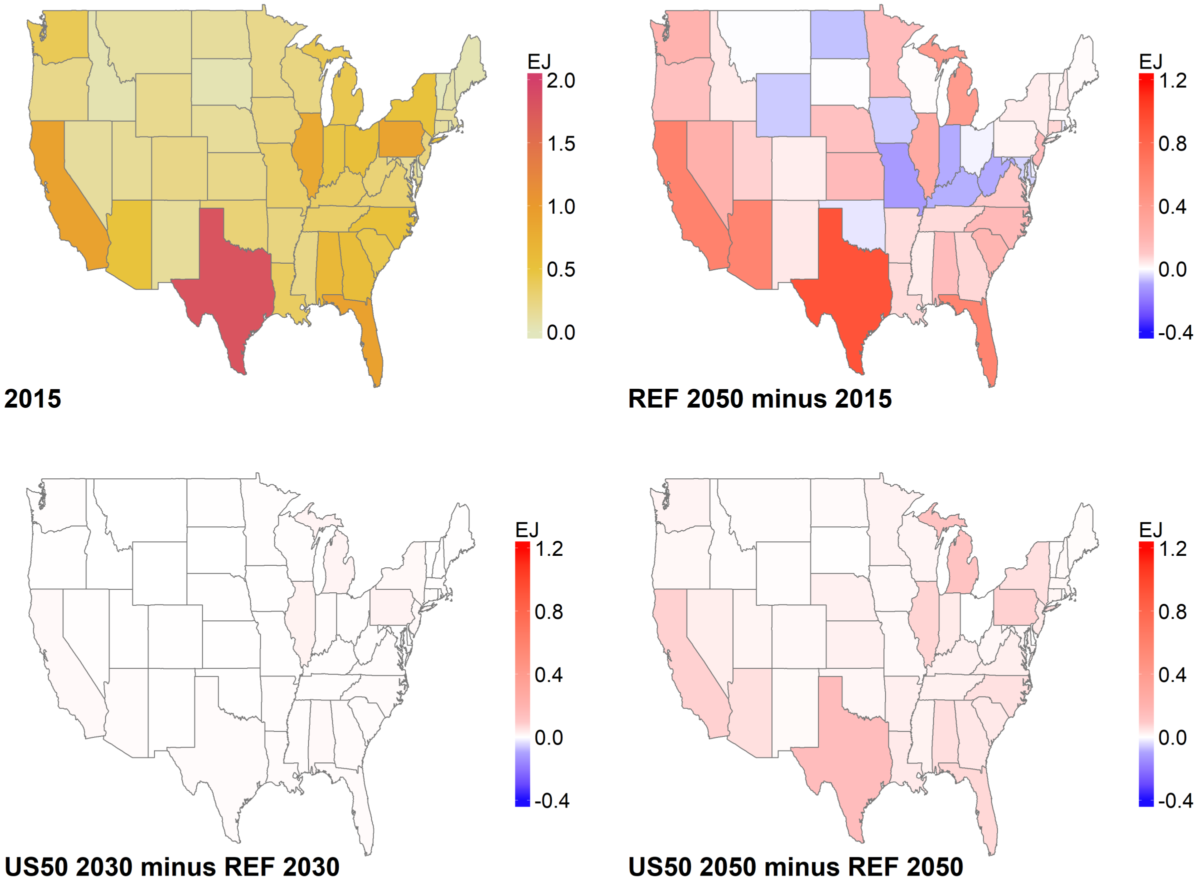


Supplementary Figure 13 Changes in electricity generation (EJ) for continental US states in REF and US50

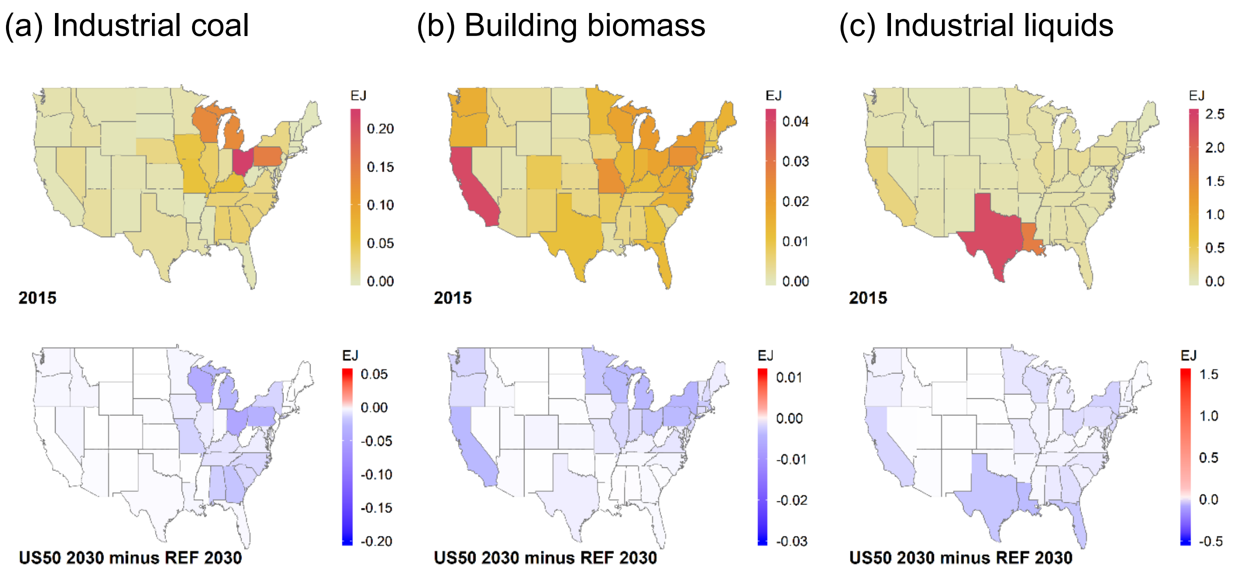


Supplementary Figure 14 Energy consumption changes for high emission intensity sources - industrial coal (a), building biomass (b), and industrial liquids (c) for continental US states in REF and US50 relative to REF in 2030.

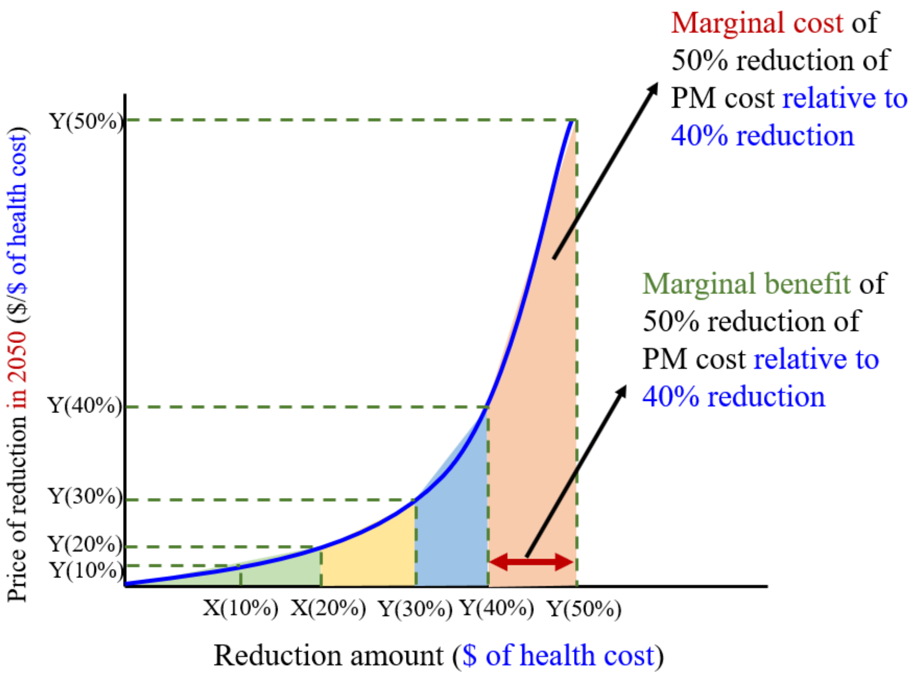


Supplementary Figure 15 Schematic of estimating policy cost of PMMC constraints in 2050 based on a numerical approach.

## Supplementary Tables

Supplementary Table 1 Modification of energy use in GCAM-USA for BASE energy trajectory

| Sector | Modification |
|---|---|
| Electricity | Harmonization of total electricity generation to Annual Energy Outlook (AEO) 2018[1] |
| | Harmonization of electricity generation from coal to AEO 2018[1] |
| | Updated wind and solar technology cost to reflect more recent market trends[2] |
| | Harmonization of electricity generation from wind and solar in 2015 and 2020 to AEO 2018[1] |
| | Updated nuclear power availability assumptions[3] |
| | Electricity technology specific requirement in CA[4] and VT[5] (no new coal in California, and no nuclear in VT) |
| Industry | Harmonization of industrial coal use to AEO 2018[1] |
| | No coal-to-liquids and gas-to-liquids in refineries[*] |
| Buildings | Harmonization of total building energy use to AEO 2018[1] |
| | Only LED and CFL bulbs allowed after 2010[6] |
| | Wood furnace efficiency improvement[7] |
| Transportation | Updated battery electricity vehicle costs[8] |

* Expert judgement

Supplementary Table 2 Modification of air pollutant emissions in GCAM-USA

| Sector | Modification |
|---|---|
| Electricity | Future-year emission factors for $NO_x$ and $SO_2$ from coal, gas, and biomass are obtained from the Integrated Planning Model (IPM)[9,10]<br>Future-year emission factors for other pollutants from other emission sources are obtained from the Greenhouse Gases Regulated Emissions, and Energy use in Transportation (GREET) model[11] |
| Industry | Base- and future-year emission factors for pollutants from industry energy use are obtained from GREET[11]<br>Base- and future-year emission factors for pollutants from refinery are obtained from the MARKet Allocation (MARKAL) energy modeling framework[12]<br>State- and pollutant-specific adjustment factors are applied to base-year emission factors to be harmonized with 2011 National Emission Inventory (NEI)[13] |
| Buildings | Updated emission factors for $PM_{2.5}$ and PM10 from residential wood combustion to reflect technology turnover under EPA regulations[7] |
| Transportation | Updated emission factors for light- and heavy-duty vehicles from results of the Motor Vehicle Emissions Simulator (MOVES) model[14]<br>Additional adjustment in lifetime and load factors of heavy-duty vehicles to be harmonized with 2011 NEI[13]<br>Future-year emission factors for $NO_x$ and $PM_{2.5}$ from marine vessels and railway cars are obtained from the NONROAD Model (Nonroad Engines, Equipment, and Vehicles)[15]<br>Future-year emission factors for $SO_2$ from domestic marine vessels are updated in accordance with marine fuel sulfur limits mandated by MARPOL Annex VI[16] |

Supplementary Table 3 Electricity generation by technology in 2050 in US50 (Unit: EJ)

| Technology | REF | US50 |
|---|---|---|
| Coal | 4.438 | 4.492 |
| Coal with CCS | 0.001 | 0.001 |
| Gas | 6.944 | 7.378 |
| Gas with CCS | 0.011 | 0.014 |
| Oil | 0.094 | 0.074 |
| Biomass | 0.257 | 0.214 |
| Biomass with CCS | 0.001 | 0.001 |
| Nuclear | 2.849 | 2.849 |
| Geothermal | 0.159 | 0.171 |
| Hydro | 0.940 | 0.940 |
| Wind | 2.042 | 2.339 |
| Solar CSP | 0.008 | 0.009 |
| Solar PV | 3.397 | 3.905 |
| CHP | 0.450 | 0.420 |
| Total | 21.591 | 22.807 |

Supplementary Table 4 Mean and median state-level PMMC coefficients in 2015 and 2050 (Unit: \$2018 tonne$^{-1}$ of pollutant emissions)

| | | Building | | Electricity | | Industry | | Transportation | |
|---|---|---|---|---|---|---|---|---|---|
| | | 2015 | 2050 | 2015 | 2050 | 2015 | 2050 | 2015 | 2050 |
| $PM_{2.5}$ | mean | 170901 | 260383 | 181623 | 277951 | 189054 | 289558 | 198129 | 303791 |
| | median | 143237 | 208046 | 150874 | 219216 | 141670 | 212189 | 160160 | 241794 |
| | s.d. | 128566 | 202900 | 161554 | 259098 | 151916 | 241867 | 153753 | 244584 |
| $SO_2$ | mean | 25589 | 45635 | 25852 | 46035 | 25785 | 45948 | 25617 | 45699 |
| | median | 23862 | 44241 | 24017 | 42739 | 25324 | 46282 | 24110 | 44318 |
| | s.d. | 9874 | 17099 | 10143 | 17518 | 9461 | 16186 | 9869 | 16933 |
| $NO_x$ | mean | 12938 | 18460 | 12122 | 17344 | 11615 | 16492 | 12548 | 17870 |
| | median | 8578 | 11925 | 9390 | 13319 | 8975 | 12767 | 8858 | 12358 |
| | s.d. | 10825 | 15831 | 10411 | 15323 | 9642 | 13919 | 10143 | 14716 |

Supplementary Table 5 State-level estimates of total policy cost and $PM_{2.5}$ health benefit in US50 in 2050 (Unit: 2018 $ Billion)

| CSAPR States | | | | Non-CSAPR states | | | |
|---|---|---|---|---|---|---|---|
| | Total policy cost | Total health benefit | benefit/cost | | Total policy cost | Total health benefit | benefit/cost |
| AL | 0.69 | 5.31 | 7.70 | AR | 0.14 | 0.59 | 4.18 |
| GA | 0.99 | 7.97 | 8.06 | AZ | 0.08 | 0.30 | 3.88 |
| IA | 0.22 | 1.21 | 5.48 | CA | 0.82 | 3.65 | 4.47 |
| IL | 0.61 | 3.92 | 6.42 | CO | 0.07 | 0.31 | 4.13 |
| IN | 0.32 | 2.20 | 6.89 | CT | 0.38 | 3.62 | 9.63 |
| KS | 0.12 | 0.56 | 4.83 | DC | 0.02 | 0.12 | 6.34 |
| KY | 0.44 | 2.20 | 5.05 | DE | 0.09 | 0.55 | 5.80 |
| MD | 0.34 | 2.98 | 8.80 | FL | 1.21 | 6.44 | 5.31 |
| MI | 1.44 | 11.45 | 7.95 | ID | 0.12 | 0.66 | 5.39 |
| MN | 0.67 | 4.61 | 6.91 | LA | 1.43 | 5.46 | 3.82 |
| MO | 0.60 | 4.05 | 6.80 | MA | 0.50 | 5.05 | 10.21 |
| NC | 0.69 | 4.62 | 6.72 | ME | 0.17 | 0.83 | 4.91 |
| NE | 0.07 | 0.35 | 4.94 | MS | 0.15 | 0.69 | 4.48 |
| NJ | 0.73 | 5.28 | 7.19 | MT | 0.00 | 0.01 | 3.64 |
| NY | 1.31 | 13.49 | 10.28 | ND | 0.00 | 0.01 | 4.31 |
| OH | 2.20 | 14.50 | 6.58 | NH | 0.29 | 2.75 | 9.53 |
| PA | 1.79 | 12.94 | 7.23 | NM | 0.01 | 0.04 | 3.42 |
| SC | 0.51 | 3.91 | 7.61 | NV | 0.13 | 0.59 | 4.63 |
| TN | 0.42 | 2.50 | 5.88 | OK | 0.17 | 0.79 | 4.57 |
| TX | 0.98 | 3.79 | 3.87 | OR | 0.43 | 2.94 | 6.77 |
| VA | 0.44 | 2.50 | 5.72 | RI | 0.07 | 0.68 | 9.46 |
| WI | 1.43 | 14.40 | 10.07 | SD | 0.01 | 0.08 | 5.27 |
| WV | 0.07 | 0.28 | 3.78 | UT | 0.02 | 0.15 | 6.11 |
| | | | | VT | 0.15 | 1.09 | 7.19 |
| | | | | WA | 0.38 | 2.15 | 5.70 |
| | | | | WY | 0.02 | 0.07 | 4.39 |

Supplementary Table 6 State-level estimates of marginal policy cost and $PM_{2.5}$ health benefit in HR US50 in 2050 (Unit: 2018 $ Billion), relative to HR REF.

| | Marginal policy cost | Marginal health benefit | | Marginal policy cost | Marginal health benefit |
|---|---|---|---|---|---|
| AL | 1.58 | 1.77 | NC | 1.57 | 1.76 |
| AR | 0.52 | 0.58 | ND | 0.01 | 0.01 |
| AZ | 0.16 | 0.18 | NE | 0.32 | 0.36 |
| CA | 3.71 | 4.16 | NH | 2.09 | 2.35 |
| CO | 0.30 | 0.34 | NJ | 4.00 | 4.49 |
| CT | 2.89 | 3.24 | NM | 0.04 | 0.05 |
| DC | 0.10 | 0.11 | NV | 0.12 | 0.14 |
| DE | 0.40 | 0.45 | NY | 8.30 | 9.31 |
| FL | 4.92 | 5.52 | OH | 8.80 | 9.87 |
| GA | 2.57 | 2.88 | OK | 0.80 | 0.90 |
| IA | 0.86 | 0.96 | OR | 2.04 | 2.29 |
| ID | 0.23 | 0.26 | PA | 7.35 | 8.25 |
| IL | 3.24 | 3.64 | RI | 0.56 | 0.63 |
| IN | 2.65 | 2.97 | SC | 1.05 | 1.18 |
| KS | 0.59 | 0.66 | SD | 0.07 | 0.08 |
| KY | 1.58 | 1.77 | TN | 0.83 | 0.93 |
| LA | 5.88 | 6.59 | TX | 4.29 | 4.81 |
| MA | 3.77 | 4.24 | UT | 0.14 | 0.16 |
| MD | 1.23 | 1.38 | VA | 1.29 | 1.45 |
| ME | 0.77 | 0.87 | VT | 0.87 | 0.97 |
| MI | 6.82 | 7.65 | WA | 1.63 | 1.83 |
| MN | 3.88 | 4.35 | WI | 4.80 | 5.38 |
| MO | 1.55 | 1.74 | WV | 0.35 | 0.40 |
| MS | 0.55 | 0.61 | WY | 0.05 | 0.05 |
| MT | 0.01 | 0.01 | **National total** | **102.12** | **114.58** |

*Both marginal policy cost and marginal health benefit in HR US50 are estimated directly relative to HR REF because HR US10 - US40 are not modelled. In this case, the marginal abatement curve is approximated to linear, so the reported marginal policy costs are overestimated.

Supplementary Table 7 State-level PMMC per thousand population from the source state (Unit: million \$ thousand$^{-1}$).

| | REF-2015 | REF-2050 | US50-2050 | | REF-2015 | REF-2050 | US50-2050 |
|---|---|---|---|---|---|---|---|
| **AL** | 1.52 | 1.55 | 0.54 | **NC** | 0.71 | 0.59 | 0.27 |
| **AR** | 0.67 | 0.50 | 0.33 | **ND** | 3.12 | 1.16 | 1.14 |
| **AZ** | 0.13 | 0.09 | 0.07 | **NE** | 1.15 | 0.66 | 0.48 |
| **CA** | 0.41 | 0.28 | 0.21 | **NH** | 2.53 | 2.13 | 0.51 |
| **CO** | 0.26 | 0.16 | 0.12 | **NJ** | 2.10 | 1.47 | 0.94 |
| **CT** | 1.79 | 1.49 | 0.52 | **NM** | 0.22 | 0.15 | 0.13 |
| **DC** | 0.83 | 0.82 | 0.56 | **NV** | 0.18 | 0.19 | 0.08 |
| **DE** | 1.45 | 0.85 | 0.35 | **NY** | 1.38 | 1.15 | 0.46 |
| **FL** | 0.45 | 0.38 | 0.19 | **OH** | 2.40 | 2.24 | 0.98 |
| **GA** | 0.95 | 0.91 | 0.32 | **OK** | 0.95 | 0.58 | 0.40 |
| **IA** | 1.31 | 0.99 | 0.58 | **OR** | 1.05 | 0.79 | 0.27 |
| **ID** | 0.45 | 0.44 | 0.16 | **PA** | 2.19 | 1.83 | 0.83 |
| **IL** | 1.33 | 0.92 | 0.63 | **RI** | 1.43 | 1.06 | 0.44 |
| **IN** | 1.93 | 1.16 | 0.85 | **SC** | 1.04 | 1.12 | 0.45 |
| **KS** | 0.97 | 0.63 | 0.44 | **SD** | 0.61 | 0.32 | 0.23 |
| **KY** | 1.89 | 1.22 | 0.76 | **TN** | 0.78 | 0.59 | 0.29 |
| **LA** | 1.92 | 2.42 | 1.30 | **TX** | 0.61 | 0.45 | 0.35 |
| **MA** | 1.65 | 1.17 | 0.46 | **UT** | 0.25 | 0.14 | 0.11 |
| **MD** | 1.18 | 0.80 | 0.40 | **VA** | 0.89 | 0.53 | 0.30 |
| **ME** | 1.53 | 1.31 | 0.71 | **VT** | 2.35 | 2.10 | 0.55 |
| **MI** | 2.25 | 1.77 | 0.65 | **WA** | 0.69 | 0.46 | 0.26 |
| **MN** | 1.76 | 1.22 | 0.53 | **WI** | 3.43 | 2.82 | 0.53 |
| **MO** | 1.60 | 1.02 | 0.43 | **WV** | 2.88 | 1.46 | 1.29 |
| **MS** | 0.70 | 0.60 | 0.38 | **WY** | 1.35 | 0.73 | 0.60 |
| **MT** | 0.39 | 0.18 | 0.17 | | | | |

* Note that population in source state is not equivalent to population exposure, which can be outside the source state.

Supplementary Table 8 Future-year (2015 to 2050) emission factors for electric sector sources in GCAM-USA (Tg $EJ^{-1}$ energy input). These emission factors represent New Source Performance Standards (NSPS) requirements.

| **Technologies** | **$NO_x$** | **$SO_2$** | **$PM_{2.5}$** |
|---|---|---|---|
| Coal (conventional pulverized) | 3.0E-02 | 2.6E-02 | Calibrated* |
| Coal (conventional pulverized with CCS) | 3.0E-02 | 2.6E-02 | 1.6E-02 |
| Coal (IGCC) | 5.6E-03 | 0.0E+00 | 3.2E-03 |
| Coal (IGCC CCS) | 5.6E-03 | 0.0E+00 | 3.2E-03 |
| Gas (CC) | 4.7E-03 | 0.0E+00 | 1.3E-04 |
| Gas (CC CCS) | 4.7E-03 | 0.0E+00 | 1.3E-04 |
| Gas (steam/CT) | 4.7E-03 | 0.0E+00 | 3.4E-03 |
| Refined liquids (CC) | 2.4E-01 | 3.1E-02 | 6.2E-03 |
| Refined liquids (CC CCS) | 2.4E-01 | 3.1E-02 | 6.2E-03 |
| Refined liquids (steam/CT) | 2.4E-01 | 3.1E-02 | 6.2E-03 |
| Biomass (conventional) | 8.6E-03 | 3.4E-02 | 3.1E-02 |
| Biomass (conventional with CCS) | 8.6E-03 | 3.4E-02 | 3.1E-02 |
| Biomass (IGCC) | 4.7E-03 | 0.0E+00 | 1.3E-04 |
| Biomass (IGCC with CCS) | 4.7E-03 | 0.0E+00 | 1.3E-04 |

* Primary $PM_{2.5}$ EFs of conventional pulverized coal plants for future years are the same as the calibrated values in 2010, differing by state

Supplementary Table 9 Future-year (2015 to 2050) emission factors for industrial sector sources in GCAM-USA (Tg $EJ^{-1}$ energy input)

| **Industrial fuel use** | | **$NO_X$** | **$SO_2$** | **$PM_{2.5}$** |
|---|---|---|---|---|
| coal/coal cogeneration | 2015 | 1.2E-01 | 5.2E-01 | 2.4E-03 |
| | 2020 | 1.2E-01 | 2.1E-01 | 2.4E-03 |
| | 2025 | 1.2E-01 | 2.1E-01 | 2.4E-03 |
| | 2030 | 1.2E-01 | 2.1E-01 | 2.4E-03 |
| | 2035 | 1.2E-01 | 2.1E-01 | 2.4E-03 |
| | 2040 | 1.2E-01 | 2.1E-01 | 2.4E-03 |
| | 2045 | 1.2E-01 | 2.1E-01 | 2.4E-03 |
| | 2050 | 1.2E-01 | 2.1E-01 | 2.4E-03 |
| gas/gas cogeneration | 2015 | 2.3E-02 | 7.1E-04 | 8.9E-03 |
| | 2020 | 2.6E-02 | 6.9E-04 | 8.8E-03 |
| | 2025 | 2.7E-02 | 6.9E-04 | 8.9E-03 |
| | 2030 | 2.9E-02 | 7.3E-04 | 9.3E-03 |
| | 2035 | 3.1E-02 | 7.5E-04 | 9.4E-03 |
| | 2040 | 3.2E-02 | 7.7E-04 | 9.6E-03 |
| | 2045 | 3.2E-02 | 7.7E-04 | 9.7E-03 |
| | 2050 | 3.3E-02 | 7.7E-04 | 9.6E-03 |
| liquid fuels/liquid fuels cogeneration | 2015 | 7.7E-02 | 2.7E-02 | 4.3E-02 |
| | 2020 | 6.3E-02 | 2.1E-02 | 3.9E-02 |
| | 2025 | 6.3E-02 | 2.1E-02 | 4.0E-02 |
| | 2030 | 6.5E-02 | 2.2E-02 | 4.8E-02 |
| | 2035 | 6.9E-02 | 2.3E-02 | 4.5E-02 |
| | 2040 | 7.5E-02 | 2.5E-02 | 4.8E-02 |
| | 2045 | 7.2E-02 | 2.5E-02 | 4.7E-02 |
| | 2050 | 7.5E-02 | 2.6E-02 | 5.0E-02 |
| biomass/ biomass cogeneration | 2015 | 2.3E-02 | 1.3E-03 | 5.0E-03 |
| | 2020 | 2.2E-02 | 1.3E-03 | 4.3E-03 |
| | 2025 | 2.0E-02 | 1.3E-03 | 3.7E-03 |
| | 2030 | 1.9E-02 | 1.3E-03 | 3.4E-03 |
| | 2035 | 1.8E-02 | 1.3E-03 | 3.1E-03 |
| | 2040 | 1.7E-02 | 1.3E-03 | 3.0E-03 |
| | 2045 | 1.6E-02 | 1.3E-03 | 2.7E-03 |
| | 2050 | 1.6E-02 | 1.3E-03 | 2.6E-03 |

Supplementary Table 10 Emission factors for building sector sources in GCAM-USA (Tg $EJ^{-1}$ energy input).

| | Technology | **$NO_X$** | **$SO_2$** | **$PM_{2.5}$** |
|---|---|---|---|---|
| Commercial | gas range | 5.3E-02 | 3.5E-03 | 6.7E-03 |
| | gas range hi-eff | 5.3E-02 | 3.5E-03 | 6.7E-03 |
| | gas cooling | 5.3E-02 | 3.5E-03 | 6.7E-03 |
| | coal furnace | 2.5E-01 | 8.6E-01 | 2.0E-02 |
| | fuel furnace | 3.0E-02 | 7.6E-03 | 3.4E-03 |
| | gas furnace | 5.3E-02 | 3.5E-03 | 6.7E-03 |
| | gas furnace hi-eff | 5.3E-02 | 3.5E-03 | 6.7E-03 |
| | wood furnace | 4.5E-02 | 5.2E-03 | 9.8E-02 |
| | fuel water heater | 3.0E-02 | 7.6E-03 | 3.4E-03 |
| | gas water heater | 5.3E-02 | 3.5E-03 | 6.7E-03 |
| | gas water heater hi-eff | 5.3E-02 | 3.5E-03 | 6.7E-03 |
| | gas | 5.3E-02 | 3.5E-03 | 6.7E-03 |
| | refined liquids | 3.0E-02 | 7.6E-03 | 3.4E-03 |
| Residential | clothes dryer | 3.7E-02 | 4.6E-04 | 3.4E-03 |
| | gas range | 3.7E-02 | 4.6E-04 | 3.4E-03 |
| | gas range hi-eff | 3.7E-02 | 4.6E-04 | 3.4E-03 |
| | LPG range | 1.2E-02 | 2.5E-02 | 1.6E-03 |
| | LPG range hi-eff | 1.2E-02 | 2.5E-02 | 1.6E-03 |
| | fuel furnace | 1.2E-02 | 2.5E-02 | 1.6E-03 |
| | fuel furnace hi-eff | 1.2E-02 | 2.5E-02 | 1.6E-03 |
| | gas furnace | 3.7E-02 | 4.6E-04 | 3.4E-03 |
| | gas furnace hi-eff | 3.7E-02 | 4.6E-04 | 3.4E-03 |
| | fuel water heater | 1.2E-02 | 2.5E-02 | 1.6E-03 |
| | fuel water heater hi-eff | 1.2E-02 | 2.5E-02 | 1.6E-03 |
| | gas water heater | 3.7E-02 | 4.6E-04 | 3.4E-03 |
| | gas water heater hi-eff | 3.7E-02 | 4.6E-04 | 3.4E-03 |
| | gas appliances | 3.7E-02 | 4.6E-04 | 3.4E-03 |
| | refined liquids | 1.2E-02 | 2.5E-02 | 1.6E-03 |
| | wood furnace - 2010 | 6.9E-02 | 9.3E-03 | 5.6E-01 |
| | wood furnace - 2015 | 6.9E-02 | 9.3E-03 | 5.1E-01 |
| | wood furnace - 2020 | 6.9E-02 | 9.3E-03 | 4.7E-01 |
| | wood furnace - 2025 | 6.9E-02 | 9.3E-03 | 4.2E-01 |
| | wood furnace - 2030 | 6.9E-02 | 9.3E-03 | 3.7E-01 |

* Only residential wood furnace technology has year specific EFs for $PM_{2.5}$ emissions

Supplementary Table 11 MACs of $NO_x$ for existing coal-fired power plants for CSAPR states.

| State | cost (1990$ $ton^{-1}$) | removal rate | State | cost (1990$ $ton^{-1}$) | removal rate |
|---|---|---|---|---|---|
| AK | 2417 | 79% | MT | 2417 | 50% |
| AL | 2417 | 31% | NC | 2417 | 31% |
| AR | 2417 | 74% | ND | 2417 | 55% |
| AZ | 2417 | 58% | NE | 2417 | 65% |
| CA | 2417 | 68% | NH | 2417 | 0% |
| CO | 2417 | 52% | NJ | 2417 | 53% |
| CT | 2417 | 66% | NM | 2417 | 55% |
| DE | 2417 | 30% | NV | 2417 | 49% |
| FL | 2417 | 55% | NY | 2417 | 49% |
| GA | 2417 | 59% | OH | 2417 | 37% |
| HI | 2417 | 79% | OK | 2417 | 68% |
| IA | 2417 | 53% | OR | 2417 | 51% |
| ID | 2417 | 67% | PA | 2417 | 37% |
| IL | 2417 | 52% | SC | 2417 | 31% |
| IN | 2417 | 46% | SD | 2417 | 79% |
| KS | 2417 | 58% | TN | 2417 | 42% |
| KY | 2417 | 40% | TX | 2417 | 43% |
| LA | 2417 | 51% | UT | 2417 | 53% |
| MA | 2417 | 24% | VA | 2417 | 41% |
| MD | 2417 | 26% | WA | 2417 | 51% |
| MI | 2417 | 50% | WI | 2417 | 46% |
| MN | 2417 | 46% | WV | 2417 | 29% |
| MO | 2417 | 49% | WY | 2417 | 56% |
| MS | 2417 | 71% | | | |

Supplementary Table 12 MACs of $SO_2$ for existing coal-fired power plants for CSAPR states.

| State | cost (1990$/ton) | removal rate | State | cost (1990$/ton) | removal rate |
|---|---|---|---|---|---|
| AK | 3802 | 95% | MT | 3802 | 51% |
| AL | 3802 | 88% | NC | 3802 | 77% |
| AR | 3802 | 95% | ND | 3802 | 93% |
| AZ | 3802 | 22% | NE | 3802 | 94% |
| CA | 3802 | 90% | NH | 3802 | 95% |
| CO | 3802 | 81% | NJ | 3802 | 42% |
| CT | 3802 | 90% | NM | 3802 | 0% |
| DE | 3802 | 95% | NV | 3802 | 79% |
| FL | 3802 | 74% | NY | 3802 | 91% |
| GA | 3802 | 90% | OH | 3802 | 87% |
| HI | 3802 | 95% | OK | 3802 | 94% |
| IA | 3802 | 92% | OR | 3802 | 95% |
| ID | 3802 | 95% | PA | 3802 | 78% |
| IL | 3802 | 94% | SC | 3802 | 78% |
| IN | 3802 | 89% | SD | 3802 | 95% |
| KS | 3802 | 85% | TN | 3802 | 92% |
| KY | 3802 | 78% | TX | 3802 | 93% |
| LA | 3802 | 95% | UT | 3802 | 75% |
| MA | 3802 | 92% | VA | 3802 | 90% |
| MD | 3802 | 74% | WA | 3802 | 0% |
| MI | 3802 | 94% | WI | 3802 | 93% |
| MN | 3802 | 80% | WV | 3802 | 69% |
| MO | 3802 | 94% | WY | 3802 | 71% |
| MS | 3802 | 94% | | | |

Supplementary Table 13 MACs of $NO_x$ for industrial coal

| State | cost(1990$/ton) | removal rate | State | cost(1990$/ton) | removal rate |
|---|---|---|---|---|---|
| AL | 4177 | 35% | MO | 3522 | 90% |
| AK | 4976 | 79% | NE | 3566 | 73% |
| AR | 1519 | 4% | NY | 1383 | 87% |
| CA | 1307 | 83% | NC | 5575 | 14% |
| CO | 3335 | 18% | ND | 3495 | 38% |
| FL | 1880 | 75% | OH | 9793 | 64% |
| GA | 2869 | 83% | OK | 2526 | 82% |
| ID | 3030 | 67% | PA | 11800 | 12% |
| IL | 4723 | 17% | SC | 5297 | 63% |
| IN | 1604 | 81% | TN | 1000 | 87% |
| IA | 2302 | 43% | TX | 58101 | 13% |
| KY | 6829 | 76% | UT | 3520 | 20% |
| MD | 624 | 88% | VA | 3258 | 41% |
| MI | 4063 | 83% | WV | 2928 | 23% |
| MN | 3562 | 21% | WI | 10396 | 1% |
| MS | 1358 | 91% | WY | 3516 | 38% |

Supplementary Table 14 MACs of $NO_x$ for industrial natural gas

| State | cost(1990$/ton) | removal rate | State | cost(1990$/ton) | removal rate |
|---|---|---|---|---|---|
| AL | 3560 | 68% | NE | 8072 | 59% |
| AK | 1340 | 98% | NV | 8135 | 17% |
| AZ | 2089 | 52% | NH | 8681 | 7% |
| AR | 3931 | 92% | NJ | 6136 | 41% |
| CA | 2157 | 15% | NM | 2495 | 87% |
| CO | 5551 | 80% | NY | 5540 | 53% |
| CT | 7785 | 23% | NC | 3962 | 48% |
| FL | 5735 | 50% | ND | 4960 | 88% |
| GA | 5045 | 74% | OH | 4424 | 43% |
| ID | 4715 | 55% | OK | 3366 | 69% |
| IL | 4732 | 54% | OR | 5252 | 30% |
| IN | 3868 | 74% | PA | 5192 | 55% |
| IA | 7698 | 11% | RI | 909 | 23% |
| KS | 5508 | 45% | SC | 8551 | 41% |
| KY | 3789 | 73% | SD | 8521 | 71% |
| LA | 4292 | 82% | TN | 4496 | 53% |
| ME | 23405 | 28% | TX | 3159 | 76% |
| MD | 15969 | 31% | UT | 4283 | 72% |
| MA | 8382 | 18% | VA | 3884 | 70% |
| MI | 3727 | 57% | WA | 6599 | 77% |
| MN | 7945 | 27% | WV | 4184 | 85% |
| MS | 3183 | 87% | WI | 8235 | 33% |
| MO | 5989 | 17% | WY | 3616 | 83% |
| MT | 2596 | 67% | | | |

Supplementary Table 15 MACs of $NO_x$ for industrial refined liquids

| State | cost(1990$/ton) | removal rate | State | cost(1990$/ton) | removal rate |
|---|---|---|---|---|---|
| AL | 10287 | 6.6% | MT | 3699 | 0.5% |
| AK | 6553 | 90.0% | NE | 6553 | 0.2% |
| AZ | 6553 | 0.4% | NV | 71644 | 1.3% |
| AR | 6553 | 0.5% | NJ | 6627 | 0.2% |
| CA | 4024 | 3.7% | NM | 6553 | 1.2% |
| CO | 6479 | 6.2% | NY | 7788 | 1.5% |
| DE | 9108 | 11.8% | NC | 8920 | 1.0% |
| FL | 10918 | 1.0% | OH | 6553 | 0.3% |
| GA | 17592 | 0.7% | OK | 6779 | 0.7% |
| HI | 6553 | 90.0% | OR | 6553 | 0.2% |
| ID | 6553 | 0.6% | PA | 10996 | 0.4% |
| IL | 6321 | 0.6% | RI | 6540 | 5.3% |
| IN | 6553 | 0.8% | SC | 6553 | 1.8% |
| KS | 6553 | 1.0% | TN | 8230 | 2.6% |
| KY | 6553 | 0.7% | TX | 6905 | 0.7% |
| LA | 7349 | 2.5% | UT | 6553 | 4.2% |
| ME | 16114 | 9.2% | VT | 2273 | 3.3% |
| MD | 7714 | 0.8% | VA | 24345 | 1.0% |
| MA | 10659 | 2.1% | WA | 33309 | 0.1% |
| MI | 6553 | 0.2% | WV | 6553 | 0.7% |
| MN | 6553 | 2.8% | WI | 6553 | 0.2% |
| MS | 6553 | 1.9% | WY | 6607 | 15.9% |

Supplementary Table 16 MACs of $SO_2$ for industrial coal

| State | cost(1990$/ton) | removal rate | State | cost(1990$/ton) | removal rate |
|---|---|---|---|---|---|
| AK | 9491 | 95% | NE | 9491 | 54% |
| AR | 3997 | 71% | NY | 9491 | 63% |
| CO | 16109 | 4% | NC | 6976 | 27% |
| FL | 23936 | 94% | ND | 9491 | 94% |
| GA | 10571 | 84% | OH | 10262 | 38% |
| ID | 15290 | 49% | OK | 21261 | 87% |
| IL | 4383 | 9% | PA | 12154 | 14% |
| IN | 9491 | 88% | SC | 4502 | 57% |
| IA | 9491 | 25% | TN | 180320 | 41% |
| KY | 9491 | 92% | VA | 9491 | 31% |
| MI | 7272 | 74% | WV | 8055 | 18% |
| MN | 120213 | 5% | WI | 23278 | 2% |
| MS | 7738 | 95% | WY | 6256 | 10% |
| MO | 9491 | 25% | | | |

Supplementary Table 17 MACs of $SO_2$ for industrial natural gas

| State | cost(1990$/ton) | removal rate | State | cost(1990$/ton) | removal rate |
|---|---|---|---|---|---|
| AL | 199925 | 84% | NE | 199925 | 81% |
| AR | 7851 | 66% | NM | 33641 | 6% |
| CA | 199925 | 3% | NC | 12246 | 78% |
| CO | 199925 | 56% | ND | 199925 | 32% |
| DE | 199925 | 70% | OK | 104237 | 14% |
| FL | 5600 | 89% | OR | 199925 | 48% |
| GA | 3557 | 94% | PA | 435599 | 12% |
| IL | 50259 | 7% | SC | 219788 | 85% |
| IN | 199925 | 72% | SD | 199925 | 91% |
| KY | 199925 | 74% | TN | 199925 | 27% |
| LA | 241836 | 26% | TX | 5653 | 82% |
| MN | 231910 | 56% | WA | 71551 | 62% |
| MS | 10306 | 31% | WI | 199925 | 47% |
| MT | 199925 | 54% | WY | 11323 | 5% |

Supplementary Table 18 MACs of $SO_2$ for industrial refined liquids

| State | cost(1990$/ton) | removal rate | State | cost(1990$/ton) | removal rate |
|---|---|---|---|---|---|
| CA | 59364 | 1% | NH | 40285 | 68% |
| DE | 64194 | 70% | NJ | 59364 | 11% |
| FL | 9823 | 2% | NC | 10633 | 58% |
| GA | 24744 | 30% | OK | 5313 | 10% |
| HI | 9705 | 95% | PA | 199925 | 2% |
| IL | 16225 | 5% | RI | 125324 | 19% |
| IN | 59364 | 61% | SC | 7696 | 20% |
| KY | 59364 | 46% | TN | 59364 | 46% |
| LA | 199925 | 2% | TX | 11297 | 73% |
| ME | 32059 | 11% | VT | 281979 | 32% |
| MD | 5338 | 16% | VA | 59768 | 11% |
| MA | 318620 | 46% | WA | 59364 | 28% |
| MI | 369755 | 5% | WV | 5197 | 45% |
| MN | 59364 | 26% | WI | 59364 | 49% |
| MS | 199925 | 21% | WY | 3279 | 75% |

Supplementary Table 19 MACs of $PM_{2.5}$ for industrial coal

| State | cost(1990$/ton) | removal rate | State | cost(1990$/ton) | removal rate |
|---|---|---|---|---|---|
| CO | 10806947 | 29% | NY | 88446 | 85% |
| FL | 70708 | 26% | NC | 44975 | 41% |
| GA | 76611 | 31% | ND | 112848 | 24% |
| ID | 82297 | 60% | OH | 47963 | 34% |
| IL | 182276 | 16% | SC | 67192 | 22% |
| IN | 88446 | 28% | TN | 338854 | 23% |
| IA | 88446 | 96% | VA | 3143839 | 46% |
| MI | 736861 | 31% | WV | 487466 | 45% |
| MN | 167080 | 46% | WY | 86780 | 72% |
| MO | 3221900 | 35% | | | |

Supplementary Table 20 MACs of $PM_{2.5}$ for industrial natural gas

| State | cost(1990$/ton) | removal rate | State | cost(1990$/ton) | removal rate |
|---|---|---|---|---|---|
| AL | 632906 | 74% | NE | 670887 | 56% |
| AR | 311023 | 50% | NJ | 18052948 | 11% |
| CA | 670887 | 1% | NY | 670887 | 41% |
| CO | 651269 | 11% | NC | 5618075 | 62% |
| FL | 276337 | 48% | ND | 573938 | 57% |
| GA | 263032 | 65% | OH | 164248 | 50% |
| IL | 64853 | 9% | OK | 804153 | 2% |
| IN | 623862 | 62% | OR | 670887 | 99% |
| IA | 670887 | 52% | PA | 456179 | 79% |
| KS | 670887 | 23% | TN | 670887 | 96% |
| KY | 503101 | 13% | TX | 187213 | 34% |
| LA | 100136 | 83% | UT | 480687 | 53% |
| MA | 670887 | 51% | VA | 670887 | 72% |
| MI | 1963202 | 19% | WA | 573938 | 42% |
| MN | 647180 | 51% | WV | 670887 | 28% |
| MS | 145244 | 35% | WY | 414590 | 43% |
| MO | 1774801 | 18% | | | |

Supplementary Table 21 MACs of $PM_{2.5}$ for industrial refined liquids

| State | cost(1990$/ton) | removal rate | State | cost(1990$/ton) | removal rate |
|---|---|---|---|---|---|
| AL | 1706523 | 3% | NY | 3234995 | 1% |
| CA | 9116 | 2% | OH | 9116 | 1% |
| GA | 162905 | 2% | SC | 9116 | 2% |
| IN | 9116 | 1% | TN | 222224 | 1% |
| LA | 30106 | 2% | UT | 9116 | 1% |
| ME | 17235 | 9% | VT | 168663 | 11% |
| MD | 3059 | 9% | VA | 1980522 | 1% |
| MA | 89810 | 9% | WV | 9116 | 2% |
| NJ | 1980522 | 2% | WY | 3638859 | 39% |

Supplementary Table 22 Changes of energy, emission and PMMC under US50 compared with REF in 2050 with and without MACs

| | All-sector | | Electricity | | Industry | | Building | | Transportation | |
|---|---|---|---|---|---|---|---|---|---|---|
| | w/ MAC | w/o MAC | w/ MACs | w/o MACs | w/ MACs | w/o MACs | w/ MACs | w/o MACs | w/ MACs | w/o MACs |
| Energy consumption | 0.0% | 0.1% | 4.2% | 4.9% | -5.2% | -6.0% | -1.1% | -1.2% | -0.1% | -0.1% |
| $PM_{2.5}$ emission | -51.5% | -55.1% | -7.7% | -11.8% | -61.3% | -64.7% | -73.4% | -78.8% | -0.1% | -0.4% |
| $SO_2$ emission | -32.1% | -39.9% | -4.6% | -18.5% | -62.0% | -64.3% | -19.4% | -21.7% | -0.5% | -0.4% |
| $NO_x$ emission | -13.2% | -15.2% | -2.8% | -8.8% | -34.1% | -37.0% | -7.2% | -7.9% | -0.1% | -0.1% |
| Total PMMC | -50.2% | -50.3% | -18.9% | -17.5% | -71.2% | -70.4% | -65.4% | -69.1% | -0.1% | -0.1% |
| PMMC from $PM_{2.5}$ | -61.9% | -63.9% | -12.7% | -18.2% | -71.4% | -72.0% | -81.6% | -86.1% | -0.1% | -0.1% |
| PMMC from $SO_2$ | -54.8% | -50.0% | -29.5% | -22.1% | -76.6% | -74.7% | -20.7% | -23.0% | -0.8% | -0.7% |
| PMMC from $NO_x$ | -20.5% | -18.6% | -10.3% | -10.5% | -59.3% | -52.5% | -8.5% | -9.3% | -0.2% | -0.1% |

Supplementary Table 23 Projected U.S. per capita income (GDP) and value of a statistical life (VSL) in all modeling years.

| Year | Per capita GDP* | VSL† |
|---|---|---|
| 2005 | 59.2 | **9.5** |
| 2010 | 58.1 | 9.3 |
| 2015 | 62.4 | 9.7 |
| 2020 | 66.5 | 10.0 |
| 2025 | 70.3 | 10.3 |
| 2030 | 74.3 | 10.6 |
| 2035 | 78.4 | 10.9 |
| 2040 | 83.6 | 11.4 |
| 2045 | 88.6 | 11.7 |
| 2050 | 94.7 | 12.2 |

* In million 2018 dollars at a Market Exchange Rate basis

† In million 2018 dollars; 2005 VSL in bold red is used as benchmark to translate VSLs in future years

Supplementary Table 24 State-level $NO_x$ emissions caps to represent CSAPR[9], emission caps for future modeling years are obtained from EPA (2015)[9].

| | **$NO_X$ (million metric tons)** | | | | | | |
|---|---|---|---|---|---|---|---|
| | 2016 | 2018 | 2020 | 2025 | 2030 | 2040 | 2050 |
| Alabama | 0.025 | 0.025 | 0.025 | 0.026 | 0.026 | 0.026 | 0.026 |
| Georgia | 0.023 | 0.023 | 0.023 | 0.027 | 0.024 | 0.024 | 0.024 |
| Illinois | 0.032 | 0.032 | 0.032 | 0.033 | 0.033 | 0.033 | 0.033 |
| Indiana | 0.087 | 0.092 | 0.090 | 0.092 | 0.077 | 0.077 | 0.077 |
| Iowa | 0.018 | 0.019 | 0.019 | 0.019 | 0.019 | 0.019 | 0.019 |
| Kansas | 0.023 | 0.023 | 0.023 | 0.023 | 0.024 | 0.024 | 0.024 |
| Kentucky | 0.068 | 0.065 | 0.063 | 0.068 | 0.066 | 0.066 | 0.066 |
| Maryland | 0.009 | 0.008 | 0.008 | 0.008 | 0.007 | 0.007 | 0.007 |
| Michigan | 0.065 | 0.063 | 0.063 | 0.062 | 0.062 | 0.062 | 0.062 |
| Minnesota | 0.021 | 0.022 | 0.022 | 0.023 | 0.023 | 0.023 | 0.023 |
| Missouri | 0.060 | 0.043 | 0.042 | 0.043 | 0.043 | 0.043 | 0.043 |
| Nebraska | 0.034 | 0.030 | 0.030 | 0.030 | 0.030 | 0.030 | 0.030 |
| New Jersey | 0.009 | 0.009 | 0.009 | 0.010 | 0.009 | 0.009 | 0.009 |
| New York | 0.016 | 0.017 | 0.016 | 0.016 | 0.014 | 0.014 | 0.014 |
| North Carolina | 0.045 | 0.045 | 0.042 | 0.045 | 0.043 | 0.043 | 0.043 |
| Ohio | 0.065 | 0.065 | 0.064 | 0.064 | 0.064 | 0.064 | 0.064 |
| Pennsylvania | 0.108 | 0.110 | 0.108 | 0.110 | 0.109 | 0.109 | 0.109 |
| South Carolina | 0.011 | 0.013 | 0.011 | 0.014 | 0.013 | 0.013 | 0.013 |
| Tennessee | 0.012 | 0.013 | 0.013 | 0.014 | 0.014 | 0.014 | 0.014 |
| Texas | 0.114 | 0.121 | 0.116 | 0.120 | 0.119 | 0.119 | 0.119 |
| Virginia | 0.021 | 0.024 | 0.022 | 0.023 | 0.021 | 0.021 | 0.021 |
| West Virginia | 0.056 | 0.056 | 0.056 | 0.057 | 0.057 | 0.057 | 0.057 |
| Wisconsin | 0.018 | 0.018 | 0.017 | 0.017 | 0.017 | 0.017 | 0.017 |

Supplementary Table 25 State-level $SO_2$ emissions caps to represent CSAPR, emission caps for future modeling years are obtained from EPA (2015)[9].

| | **$SO_2$ (million metric tons)** | | | | | | |
|---|---|---|---|---|---|---|---|
| | 2016 | 2018 | 2020 | 2025 | 2030 | 2040 | 2050 |
| Alabama | 0.034 | 0.037 | 0.038 | 0.039 | 0.039 | 0.039 | 0.039 |
| Georgia | 0.021 | 0.024 | 0.025 | 0.026 | 0.026 | 0.026 | 0.026 |
| Illinois | 0.044 | 0.047 | 0.047 | 0.048 | 0.050 | 0.050 | 0.050 |
| Indiana | 0.106 | 0.118 | 0.118 | 0.128 | 0.116 | 0.116 | 0.116 |
| Iowa | 0.013 | 0.013 | 0.013 | 0.013 | 0.013 | 0.013 | 0.013 |
| Kansas | 0.013 | 0.013 | 0.013 | 0.013 | 0.013 | 0.013 | 0.013 |
| Kentucky | 0.082 | 0.075 | 0.074 | 0.090 | 0.086 | 0.086 | 0.086 |
| Maryland | 0.004 | 0.005 | 0.004 | 0.007 | 0.006 | 0.006 | 0.006 |
| Michigan | 0.077 | 0.077 | 0.077 | 0.080 | 0.079 | 0.079 | 0.079 |
| Minnesota | 0.013 | 0.013 | 0.013 | 0.013 | 0.013 | 0.013 | 0.013 |
| Missouri | 0.059 | 0.063 | 0.069 | 0.073 | 0.090 | 0.090 | 0.090 |
| Nebraska | 0.022 | 0.023 | 0.023 | 0.023 | 0.028 | 0.028 | 0.028 |
| New Jersey | 0.006 | 0.006 | 0.006 | 0.006 | 0.006 | 0.006 | 0.006 |
| New York | 0.004 | 0.005 | 0.005 | 0.005 | 0.004 | 0.004 | 0.004 |
| North Carolina | 0.027 | 0.028 | 0.026 | 0.030 | 0.031 | 0.031 | 0.031 |
| Ohio | 0.099 | 0.103 | 0.104 | 0.107 | 0.102 | 0.102 | 0.102 |
| Pennsylvania | 0.056 | 0.057 | 0.056 | 0.061 | 0.058 | 0.058 | 0.058 |
| South Carolina | 0.016 | 0.023 | 0.021 | 0.027 | 0.026 | 0.026 | 0.026 |
| Tennessee | 0.036 | 0.038 | 0.038 | 0.036 | 0.038 | 0.038 | 0.038 |
| Texas | 0.131 | 0.126 | 0.119 | 0.129 | 0.127 | 0.127 | 0.127 |
| Virginia | 0.013 | 0.015 | 0.014 | 0.015 | 0.015 | 0.015 | 0.015 |
| West Virginia | 0.068 | 0.068 | 0.066 | 0.068 | 0.065 | 0.065 | 0.065 |
| Wisconsin | 0.014 | 0.014 | 0.014 | 0.014 | 0.014 | 0.014 | 0.014 |

Supplementary Table 26 CAFE Requirements in the GCAM-USA (Unit: Mileage per Gallon)

| **2015** | **2020** | **2025** | **2030** | **2035** | **2040** | **2045** | **2050** |
|---|---|---|---|---|---|---|---|
| 25.8 | 30.6 | 37.7 | 38.3 | 38.7 | 38.9 | 57.1 | 75.3 |

[1] GCAM-USA represents the Corporate Average Fuel Economy (CAFE) standards by increasing the fuel economy of new light-duty vehicles to meet specific fleet efficiency requirements at state level[13].

Supplementary Table 27 Capital cost assumptions for electricity generation technologies (2018$ $kW^{-1}$).

| Technologies* | BASE† | | | HR‡ | | |
|---|---|---|---|---|---|---|
| | **2015** | **2030** | **2050** | **2015** | **2030** | **2050** |
| Wind | 2433 | 1747 | 1737 | 1985 | 1513 | 1532 |
| PV | 2970 | 1051 | 929 | 1877 | 626 | 374 |

* All technologies not presented are assumed to be the same as GCAM-USA default values

† Adopted from Iyer et al (2017)[2] - advanced technology scenario cost assumptions

‡ Alternative wind capital cost assumptions are developed from Wiser & Bolinger (2017)[17], and alternative utility-scale solar PV capital cost assumptions are developed from Bolinger & Seel (2016)[18].

**Supplementary References**


1. US Energy Information Administration (EIA). (2018). Annual Energy Outlook 2018. Available at: https://www.eia.gov/outlooks/aeo/. [accessed Nov 2018]

2. Iyer, G., Ledna, C., Clarke, L.E., McJeon, H., Edmonds, J., and Wise, M. (2017). GCAM-USA Analysis of US Electric Power Sector Transitions, Pacific Northwest National Laboratory, Richland, Washington.

3. S&P Global Platts. (2018). No new nuclear units will be built in US due to high cost: Exelon official. Available at: https://www.spglobal.com/platts/en/market-insights/latest-news/electric-power/041218-no-new-nuclear-units-will-be-built-in-us-due-to-high-cost-exelon-official. [accessed Jan 2019]

4. Senate Bill No. 100 (SB-100), California Renewables Portfolio Standard Program: emissions of greenhouse gases. 2017-2018.

5. Vermont Department of Public Service (DPS). (2016). Comprehensive Energy Plan 2016, Executive Summary. Available at: https://publicservice.vermont.gov/sites/dps/files/documents/Pubs_Plans_Reports/State_Plans/Comp_Energy_Plan/2015/2016CEP_ES_Final.pdf [accessed Jan 2019]

6. H.R.6 – Energy Independence and Security Act of 2007. 110th Congress (2007-2008)

7. US Environmental Protection Agency (EPA). (2015). 40 CFR Parts 60. Standards of Performance for New Residential Wood Heaters, New Residential Hydronic Heaters and Forced-Air Furnaces. Fed. Register, 2015, Vol. 80, No. 50, 13672-13753.

8. Union of Concerned Scientists (UCS). (2017). Going from pump to plug: Adding Up the Savings from Electric Vehicles (EVs). Available at: https://www.ucsusa.org/clean-vehicles/electric-vehicles/ev-fuel-savings [accessed Jul 2019]

9. U.S. EPA. (2015). Power sector modeling platform v5.14: inremental documentation for v5.14. Available at: https://www.epa.gov/airmarkets/power-sector-modeling-platform-v514 [accessed Jul 2019]

10. U.S. EPA. (2013). Documentation for EPA base case v.5.13: using the integrated planning model. EPA report 450R13002. Washington, DC: Office of Air and Radiation; 2013. 355 pp.

11. Argonne National Laboratory (ANL). (2014). Criteria air pollutant and greenhouse gas emission factors compiled by eastern research group for incorporation in GREET. Systems Assessment Group, Energy Systems Division. Available at https://greet.es.anl.gov/files/emission-factors-2014 [accessed Jul 2019].

12. Lenox, C. S., Dodder, R. S,, Gage, C. L,, Kaplan, P. O., Loughlin, D. H., Yelverton, W. (2013). EPA U.S. nine-region MARKAL database: database documentation. EPA600/B-13/203. NC: Office of Research and Development, U.S. Environmental Protection Agency, Research Triangle Park.

13. Shi, W., Ou, Y., Smith, S. J., Ledna, C. M., Nolte, C. G., & Loughlin, D. H. (2017). Projecting state-level air pollutant emissions using an integrated assessment model: GCAM-USA. Applied energy, 208, 511-521.

14. U.S. EPA. (2014). MOVES (motor vehicle emission simulator); Available at: https://www3.epa.gov/otaq/models/moves/ [accessed Nov, 2016].

15. U.S. EPA. (2008). NONROAD Model (Nonroad Engines, Equipment, and Vehicles). Available at: https://19january2017snapshot.epa.gov/moves/nonroad-model-nonroad-engines-equipment-and-vehicles_.html [accessed Jul, 2019].

16. International Maritime Organization (IMO). (2017). Sulphur oxides (SO2) – regulation 14. Available at: http://www.imo.org/en/OurWork/Environment/PollutionPrevention/AirPollution/Pages/Sulphur-oxides-(SOx)-%E2%80%93- Regulation-14.aspx [accessed March 7, 2017].

17. Wiser, R., Bolinger, M. 2016 Wind Technologies Market Report. U.S. Department of Energy. Prepared by the Lawrence Berkeley National Laboratory (LBNL). DOE/GO-102917-5033. (2017).

18. Bolinger, M., Seel, J. Utility-Scale Solar 2015 – An empirical analysis of project cost, performance, and pricing trends in the United States. U.S. Department of Energy SunShot. Prepared by the Lawrence Berkeley National Laboratory (LBNL). LBNL-1006037. (2016).
