# Supplementary material for: Air pollution control strategies directly limiting national health damages in the US
Source: Nat Commun. 2020 Feb 19;11:957. doi: 10.1038/s41467-020-14783-2 (PMC7031358; doi:10.1038/s41467-020-14783-2)
Supplement: Supplementary file 3 — Reporting Summary [file 41467_2020_14783_MOESM3_ESM.pdf]

nature research

Corresponding author(s): Daniel H. Loughlin

Last updated by author(s): Feb 3, 2020

Reporting Summary

Nature Research wishes to improve the reproducibility of the work that we publish. This form provides structure for consistency and transparency in reporting. For further information on Nature Research policies, seeAuthors & Referees and theEditorial Policy Checklist .

Statistics

For all statistical analyses, confirm that the following items are present in the figure legend, table legend, main text, or Methods section.

n/a	Confirmed	
☐	☒	The exact sample size (*n*) for each experimental group/condition, given as a discrete number and unit of measurement
☒	☐	A statement on whether measurements were taken from distinct samples or whether the same sample was measured repeatedly
☒	☐	The statistical test(s) used AND whether they are one- or two-sided<br>*Only common tests should be described solely by name; describe more complex techniques in the Methods section.*
☒	☐	A description of all covariates tested
☒	☐	A description of any assumptions or corrections, such as tests of normality and adjustment for multiple comparisons
☐	☒	A full description of the statistical parameters including central tendency (e.g. means) or other basic estimates (e.g. regression coefficient) AND variation (e.g. standard deviation) or associated estimates of uncertainty (e.g. confidence intervals)
☒	☐	For null hypothesis testing, the test statistic (e.g. *F*, *t*, *r*) with confidence intervals, effect sizes, degrees of freedom and *P* value noted<br>*Give P values as exact values whenever suitable.*
☒	☐	For Bayesian analysis, information on the choice of priors and Markov chain Monte Carlo settings
☒	☐	For hierarchical and complex designs, identification of the appropriate level for tests and full reporting of outcomes
☒	☐	Estimates of effect sizes (e.g. Cohen's *d*, Pearson's *r*), indicating how they were calculated

Our web collection on statistics for biologists contains articles on many of the points above.

Software and code

Policy information about availability of computer code

Data collection	We generated data for this study we modified version 4.3 of the Global Change Assessment Model with U.S. state-level resolution (GCAM-USA). GCAM-USA is publicly available from Pacific Northwest National Laboratories. Our modifications are available upon request.
Data analysis	Data analysis was accomplished using the R software package and with Microsoft Excel. R files are available upon request.

For manuscripts utilizing custom algorithms or software that are central to the research but not yet described in published literature, software must be made available to editors/reviewers. We strongly encourage code deposition in a community repository (e.g. GitHub). See the Nature Research guidelines for submitting code & software for further information.

Data

Policy information about availability of data

All manuscripts must include a data availability statement. This statement should provide the following information, where applicable:
- Accession codes, unique identifiers, or web links for publicly available datasets
- A list of figures that have associated raw data
- A description of any restrictions on data availability

Data used to perform this study can be found in the Supplementary Information and Supplementary Data. GCAM-USA is publicly available (https://github.com/JGCRI/gcam-core/releases). The additional GCAM-USA input files required to perform this study are available from the corresponding author upon request.

Field-specific reporting

Please select the one below that is the best fit for your research. If you are not sure, read the appropriate sections before making your selection.

Ecological, evolutionary & environmental sciences study design

All studies must disclose on these points even when the disclosure is negative.

Study description	An application of the GCAM-USA human earth system model in which we evaluate several scenarios in which the mortality costs associated with fine particulate matter are constrained at various levels. We then identify the cost-effective technological pathways and develop insights regarding which technologies yield the greatest health benefits.
Research sample	GCAM-USA is a deterministic model, so we do not perform any sampling, per se. However, there is uncertainty in factors such as the role of renewable energy in the future. We address this uncertainty by evaluating an alternative scenario with lower cost renewables.
Sampling strategy	No sampling occurred.
Data collection	Dr. Yang Ou performed the modeling experiments and extracted GCAM-USA results from the model's output database.
Timing and spatial scale	We used GCAM-USA to produce U.S. state-level outputs at 5-year timesteps, extending from 2010 through 2050. Constraints on mortality costs were applied nationally, and the model was allowed to apportion those constraints to states, sectors, and pollutants in the most cost-effective manner for each modeled time period. GCAM-USA does not have foresight, so these decisions were made without considering the level of the constraint into the future.
Data exclusions	Data were not excluded.
Reproducibility	Our results are reproducible provided the modelers use GCAM-USA version 4.3 and include the model updates that we have developed. These model updates are available from the corresponding author.
Randomization	Randomization is not applicable.
Blinding	Blinding is not applicable.

Did the study involve field work? ☐ Yes ☒ No

Reporting for specific materials, systems and methods

We require information from authors about some types of materials, experimental systems and methods used in many studies. Here, indicate whether each material, system or method listed is relevant to your study. If you are not sure if a list item applies to your research, read the appropriate section before selecting a response.

Materials & experimental systems

n/a	Involved in the study	
☒	☐	Antibodies
☒	☐	Eukaryotic cell lines
☒	☐	Palaeontology
☒	☐	Animals and other organisms
☒	☐	Human research participants
☒	☐	Clinical data

Methods

n/a	Involved in the study	
☒	☐	ChIP-seq
☒	☐	Flow cytometry
☒	☐	MRI-based neuroimaging
