# Supplementary material for: Air pollution control strategies directly limiting national health damages in the US
Source: Nat Commun. 2020 Feb 19;11:957. doi: 10.1038/s41467-020-14783-2 (PMC7031358; doi:10.1038/s41467-020-14783-2)
Supplement: Supplementary file 4 — Source Data [file 41467_2020_14783_MOESM4_ESM.zip › Source Data/Description of Source Data.docx]

**Source data files for main figures and supplementary figures**

Ou et al.

Figure 1-6.csv files provide original data used to generate Main figures 1-6, respectively. Note that Figure 4 and 5 require additional processing using R files, which are available upon request.

Figure S1-S14.csv files provide original data used to generate Supplementary Figures 1-14, respectively. Some Supplementary Figures require additional R codes, which have been noted in the corresponding csv files. Supplementary Figure 15 is an illustrative scheme without actual source data.
